# A re-appraisal of mesenchymal-epithelial transition (MET) in endometrial epithelial remodeling

**DOI:** 10.1007/s00441-022-03711-z

**Published:** 2022-11-19

**Authors:** Madelyn Spooner-Harris, Karl Kerns, Michal Zigo, Peter Sutovsky, Ahmed Balboula, Amanda L. Patterson

**Affiliations:** 1 Division of Animal Sciences, University of Missouri, Columbia, MO 65211, USA; 2 Department of Obstetrics, Gynecology and Women’s Health, University of Missouri, Columbia, MO 65211, USA; 3 Department of Animal Science, Iowa State University, Ames, IA 50011, USA

**Keywords:** Endometrial epithelial remodeling, Mesenchymal-epithelial transition (MET), Endometrial regeneration, Postnatal uterine maturation, Epithelial repair

## Abstract

Mesenchymal-epithelial transition (MET) is a mechanism of endometrial epithelial regeneration. It is also implicated in adenocarcinoma and endometriosis. Little is known about this process in normal uterine physiology. Previously, using pregnancy and menses-like mouse models, MET occurred only as an epithelial damage/repair mechanism. Here, we hypothesized that MET also occurs in other physiological endometrial remodeling events, outside of damage/repair, such as during the estrous cycle and adenogenesis (gland development). To investigate this, *Amhr2-Cre-YFP/GFP* mesenchyme-specific reporter mice were used to track the fate of mesenchymal-derived (MD) cells. Using EpCAM (epithelial marker), EpCAM^+^YFP^+^ MD-epithelial cells were identified in all stages of the estrous cycle except diestrus, in both postpartum and virgin mice. EpCAM^+^YFP^+^ MD-epithelial cells comprised up to 80% of the epithelia during estrogen-dominant proestrus and significantly declined to indistinguishable from control uteri in diestrus, suggesting MET is hormonally regulated. MD-epithelial cells were also identified during postnatal epithelial remodeling. MET occurred immediately after birth at postnatal day (P) 0.5 with EpCAM^+^GFP^+^ cells ranging from negligible (0.21%) to 82% of the epithelia. EpCAM^+^GFP^+^ MD-epithelial cells declined during initiation of adenogenesis (P8, avg. 1.75%) and then increased during gland morphogenesis (P14, avg. 10%). MD-epithelial cells expressed markers in common with non-MD-epithelial cells (e.g., EpCAM, FOXA2, ESR1, PGR). However, MD-epithelial cells were differentially regulated postnatally and in adults, suggesting a functional distinction in the two populations. We conclude that MET occurs not only as an epithelial damage/repair mechanism but also during other epithelial remodeling events, which to our knowledge has not been demonstrated in other tissues.

## Introduction

The adult uterus is a dynamic organ that responds to synchronized hormonal (e.g., estrogen and progesterone) changes throughout the menstrual cycle in women and estrous cycle in other mammals. These cycles are characterized by cellular growth, differentiation, degeneration, and regeneration with the extent of degeneration and regeneration differing by species. In women, two-thirds of the endometrium (uterine lining) is shed and regenerated approximately 400 times from puberty to menopause. In estrous cycling species, such as mice, the endometrium is not shed; however, it responds similarly to patterns of ovarian hormones resulting in cellular turnover by proliferation and apoptosis (Wood et al. 2007). In species with invasively implanting embryos, including humans and mice, the endometrium is extensively remodeled during pregnancy and regenerated postpartum. Proper endometrial regeneration is necessary for the preparation of the uterus for subsequent reproductive cycles and pregnancy.

The two primary hormonally responsive cell types that comprise the endometrium are stromal-mesenchymal cells and epithelial cells [luminal epithelium (LE) and glandular epithelium (GE)]. These two cell types are etiologically, phenotypically, and functionally distinct and both are subject to degeneration and regeneration. Epithelial regeneration is likely facilitated by multiple processes to ensure swift repair of the luminal barrier (LE) to protect against infection and hemorrhage and further to regenerate the endometrial glands (GE) that are necessary for optimal fertility (Kelleher et al. 2019; Spooner et al. 2021). The most widely accepted mechanism is the contribution of epithelial stem/progenitor cells. Increasing evidence suggests a novel mechanism, mesenchymal-epithelial transition (MET), contributes to endometrial epithelial regeneration during menstruation and postpartum (Garry et al. 2009; Huang et al. 2012; Patterson et al. 2013; Cousins et al. 2014; Yin et al. 2019).

MET and its counterpart, epithelial-mesenchymal transition (EMT), are cellular mechanisms important in embryonic development but may become dysregulated and contribute to diseases like cancer and endometriosis (Matsuzaki and Darcha 2012; Bartley et al. 2014; Banyard and Bielenberg 2015; Wilson et al. 2020). Studies have demonstrated that MET occurred in the endometrium following parturition and induced endometrial shedding in mice (Huang et al. 2012; Patterson et al. 2013; Yin et al. 2019). In two seminal studies, lineage-tracing was performed using Anti-Mullerian Hormone Receptor Type 2 *(Amhr2)-Cre* mice to drive expression of either EYFP (*Amhr2-Cre;Rosa26-Eyfp*) (Patterson et al. 2013) or *LacZ* (*Amhr2-Cre;Rosa26-LacZ*) (Huang et al. 2012) reporters. The *Amhr2* promoter is active only in mesenchymal cells of the uterus (i.e., stroma and myometrium) (Jamin et al. 2002; Arango et al. 2008; Huang et al. 2012; Patterson et al. 2013; Saatcioglu et al. 2019); therefore, reporter expression was restricted to mesenchymal cells and mesenchymal-derived (MD) cells. In immature and virgin mice, reporter expression was shown to be restricted to the mesenchyme. However, following completed endometrial regeneration postpartum, EYFP^+^ or *LacZ*^+^ MD cells were identified in the GE and LE suggesting that stromal-mesenchymal cells underwent MET as a mechanism of epithelial regeneration (Huang et al. 2012; Patterson et al. 2013). Furthermore, dual staining identified cells co-expressing stromal and epithelial markers during menses-like endometrial regeneration, identifying putative mesenchymal-epithelial transitional cells (Patterson et al. 2013; Cousins et al. 2014). It is probable that MET occurs monthly in women during menstruation. Although studies are scarce, this was proposed as early as 1897 (Heape 1897). Notably, histological techniques and scanning electron microscopy were used to evaluate menstrual epithelial regeneration, revealing possible contribution of stromal cells to the LE, presumably through MET (Baggish et al. 1967; Garry et al. 2009). Together, these studies suggest that MET is a mechanism for epithelial regeneration during menstruation and postpartum, though little is known about this process.

Although MET appears to be a mechanism of epithelial regeneration following endometrial damage in menstruation and pregnancy, its role in other epithelial remodeling events is unknown. There are three primary physiological epithelial remodeling events that occur in the uterus. The first begins after birth when the immature single layer of epithelium (LE) differentiates and invaginates into the stroma forming endometrial glands (GE) in a process termed adenogenesis (Spencer et al. 2005). This is a damage/repair-independent process. The second occurs cyclically in sexually mature adult females. This is the estrous cycle (or in women, the menstrual cycle), during which ovarian estradiol (E_2_) and progesterone (P_4_) regulate epithelial proliferation and apoptosis (Wood et al. 2007). The estrous cycle, in contrast to the menstrual cycle, is damage/repair independent as there is not endometrial shedding. The third event occurs during pregnancy and postpartum and the extent of remodeling is species dependent. In pregnancy, the epithelium is remodeled during embryo implantation, and postpartum is repaired and/or regenerated as the uterus returns to its pre-pregnant state. Currently, it is unknown if stromal cells contribute to epithelial remodeling, via MET, under non-damage/repair conditions in postnatal adenogenesis and the estrous cycle.

We hypothesize that MET occurs during all three epithelial remodeling events including in non-damage/repair conditions. Therefore, the objectives of the current study were to (1) characterize mesenchymal-derived (MD) epithelial cells in postpartum uteri, (2) investigate MET in epithelial remodeling during the estrous cycle, and (3) evaluate the role of MET in postnatal epithelial maturation and adenogenesis.

## Materials and methods

### Animals

*Amhr2-Cre* mice (Jamin et al. 2002) were obtained from the Mutant Mouse Resource and Research Center, *Rosa26-Stop*^*fl/fl-EYFP*^ (Srinivas et al. 2001) (Jax stock #: 006148), *Rosa26-Stop*^*fl/fl-tT*^ (Wang et al. 2008) (Jax stock #: 008603), and *TRE-H2B-GFP* (Tumbar et al. 2004) (Jax stock #: 005104) mice were obtained from Jackson Laboratories.

### Lineage-tracing studies

**Fig. 1:**
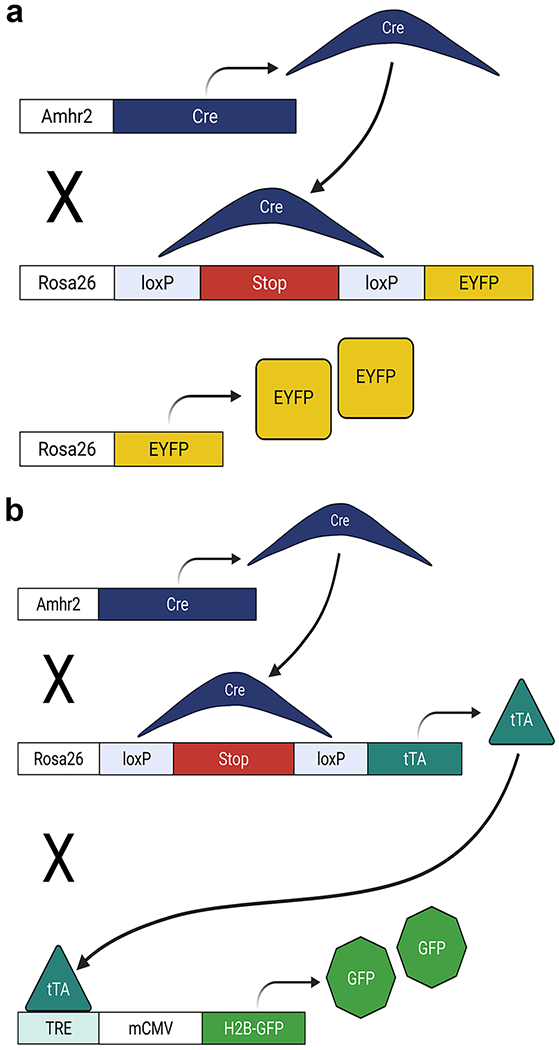
Schematic diagrams of lineage-tracing mouse models. **a** Mice expressing Cre under the control of the anti-Müllerian Hormone Type II Receptor (Amhr2) promoter (*Amhr2-Cre*), were crossed to mice with a loxP-floxed STOP sequence upstream of enhanced yellow fluorescent protein (*EYFP*) (*Rosa26-Stop^fl/fl–EYFP^*) in the Rosa locus. Upon Cre mediated excision of the STOP sequence, EYFP was constitutively expressed in mesenchymal and mesenchymal-derived cells within the uterus of double transgenic mice (*Amhr2-Cre; Rosa26-EYFP)*. **b** *Amhr2-Cre* mice were crossed to mice with a loxP-floxed STOP sequence upstream of the tetracycline trans-activator (tTA) (*Rosa26-Stop^fl/fl–tTA^*), resulting in constitutive expression of tTA. A third cross was made with mice that had a histone *H2bj-GFP* fusion gene controlled by an up-stream tetracycline-inducible promoter (TRE, tetracycline response element) (*TRE-H2b-GFP*) resulting in triple transgenic mice (*Amhr2-Cre; Rosa26-tTA; H2B-GFP*). In *Amhr2-Cre; Rosa26-tTA; H2B-GFP* mice, tTA bound the TRE,inducing expression of H2B-GFP that was incorporated into nucleosomes serving to label mesenchymal and mesenchymal-derived cells within the uterus

*Amhr2-Cre* mice were crossed to *Rosa26-Stop*^*fl/fl-EYFP*^ mice to generate double transgenic reporter females (*Amhr2-Cre; Rosa26-EYFP*) and littermate controls (*Amhr2-WT; Rosa26-Stop*^*fl/fl-EYFP*^). In cells with *Amhr2* promotor activity (mesenchymal cells-stroma and myometrium), Cre-mediated excision of the loxP-floxed stop codon in the *Rosa26* locus resulted in constitutive expression of EYFP (Fig. 1a). Uteri from *Amhr2-Cre; Rosa26-EYFP* and control females were collected at the following time points: postnatal (P) day 21 (*n* = 4), and sexually mature virgin adults (2–5 months) in proestrus (*n* = 7), estrus (*n* = 4), metestrus (*n* = 10), and diestrus (*n* = 4). For postpartum time points, after one pregnancy per mouse, uteri were collected at 96 h (*n* = 4), 2 weeks (*n* = 5), 3 weeks (*n* = 9), 2 months (*n* = 4), and 3 months (*n* = 3). Uteri were observed grossly and the number of implantation sites per mouse was counted. Postpartum mice were also staged at the time of collection resulting in the following: proestrus (*n* = 4), estrus (*n* = 4), metestrus (*n* = 5), and diestrus (*n* = 8).

To generate triple transgenic females (*Amhr2-Cre; Rosa26-tTA; H2B-GFP*), *Rosa26-Stop^fl/fl-tTA^* mice were crossed with *TRE-H2B-GFP* reporter mice to produce homozygous, double transgenic mice (*Rosa26-Stop^fl/fl-tTA^; H2B-GFP*) before a final cross with *Amhr2-Cre* mice (Fig. 1b). *Amhr2-WT; Rosa26-Stop^fl/fl-tTA^; H2B-GFP* mice served as controls. In triple transgenic mice (*Amhr2-Cre; Rosa26-tTA; H2B-GFP*), H2B-GFP was constitutively expressed and incorporated into nucleosomes in cells with *Amhr2* promotor activity and any daughter cells. This resulted from Cre-mediated excision of the loxP-floxed stop codon in the *Rosa26* locus leading to expression of tTA that then bound to TRE driving H2B-GFP expression. Uteri were collected at P0.5 (*n* = 5), P3 (*n* = 4), P8 (*n* = 6), P14 (*n* = 6), and P21 (*n* = 5).

### Estrous cycle staging

Cycle staging was ascertained using vaginal lavage method with 40 μL of 10% trypan blue/PBS solution. Stage was determined microscopically according to proportions of cornified epithelial cells, nucleated epithelial cells, and leukocytes (Ajayi and Akhigbe 2020) and verified by gross visualization of the uterus (Bertolin and Murphy 2014).

### Uterine epithelial cell isolation and image-based flow cytometry

Uterine epithelial cell isolation was performed using a modified protocol (De Clercq et al. 2017). Uterine horns were collected from *Amhr2-Cre; Rosa26-EYFP, Amhr2-Cre; Rosa26-tTA; H2B-GFP*, and control females, cut longitudinally to expose the lumen, cut into 2–4-mm pieces and placed in a 15-mL conical tube with HBSS + (Hank’s Balanced Salt Solution with antibiotic/antimycotics, Gibco, Waltham, MA) and 0.25% trypsin (Sigma, St. Louis, MO). Adult uteri underwent a series of digestions: 4 °C oscillating at 50–70 rpm for 1 h, RT without oscillation for 40 min, and 37 °C without oscillation for 25 min. Postnatal uteri underwent a similar series of digestions: RT without oscillation for 12 min (P 0.5), 17 min (P 3), 20 min (P 8), 25 min (P 14), or 30 min (P 21), 37 °C without oscillation for 5 min (P 0.5), 10 min (P 3, P 8), 15 min (P 14), or 20 min (P 21). All contents were poured over a 100 μm cell strainer and collected in a 50-mL conical tube. Uterine pieces were transferred into Mouse Endometrial Epithelial Cell (MEEC) media containing 10% FBS for 5 min to deactivate trypsin and then into HBSS + in a 15-mL conical tube and vortexed vigorously for 10 s to release epithelial cells. Cells were combined with previously strained contents. Transfer of tissue into MEEC media, vortex, and filtration were repeated three times and cells were pooled. Cell suspensions (adult uteri only) were cleared of red blood cells with AKC lysis buffer (Gibco) and then incubated in PBS buffer (1 × PBS, 2% FBS, 1%BSA, 1 mM EDTA) for 10 min. Cells were pelleted and then stained with EpCAM-APC (1:10; BD Biosciences, San Jose, CA; cat #563478) in PBS buffer for 15 min at RT. After a final wash and resuspension with PBS, buffer cells were analyzed by Amnis FlowSight image-based flow cytometer (Kennedy et al. 2014; Kerns et al. 2018) (Luminex, Austin, TX), fitted with a 20 × microscope objective, using FlowSight dedicated IDEAS software (Version 6.2, Luminex). Events were gated for singlets [bright field (BF)-aspect ratio by BF-area] (SFig. 1a), then side scatter by EpCAM-APC fluorescence to identify epithelial cells (SFig. 1a’), and finally EpCAM-APC by YFP/GFP fluorescence to visualize EpCAM^+^YFP/GFP^−^ and EpCAM^+^YFP/GFP^+^ populations (SFig. 1a”). EpCAM and YFP/GFP expression was confirmed in single cell images acquired using the FlowSight 20 × objective (SFig. 1b) and in tissue sections by immunofluorescence for EpCAM and direct fluorescence for YFP/GFP (SFig. 1c–f).

### Gelatin embedding and frozen tissue preparation

Uterine horns were collected from *Amhr2-Cre; Rosa26-EYFP, Amhr2-Cre; Rosa26-tTA; H2B-GFP*, and control females and fixed in 8% PFA for 30 min at 4 °C, washed 3 times with ice-cold PBS and incubated in 15% sucrose buffered in PBS overnight. Samples were incubated in gelatin (7.5% gelatin, 15% sucrose in PBS; gelatin from porcine skin, Sigma) for 1 h at 37 °C, embedded in gelatin, frozen at − 50 to − 65 °C in 2-methylbutane cooled by liquid nitrogen, and stored at °80 °C until cryo-sectioning. Tissues were cryo-sectioned at 5–8 μm and thaw-mounted.

### Immunofluorescence

Gelatin was removed from thaw-mounted tissues in 37 °C PBS. The following antibodies were used for immunofluorescence: EpCAM (1:100; BD Biosciences, cat # 552370), Ki67 (1:100; Invitrogen, Waltham, MA, cat # MA5-14520), estrogen receptor alpha (ESR1; 1:100; Santa Cruz Biotechnology, Dallas, TX, cat # A0716), progesterone receptor (PGR; 1:100; Invitrogen, cat # MA5-14505), FOXA2 (1:100; Abcam, Cambridge, MA, cat # ab108422), and GFP (1:1000; Invitrogen, cat # A-11122). Tissues were blocked in PBS buffer (1 × PBS, 1% BSA, 10% normal goat serum, and 0.1% Triton X-100) for 1 h at RT before incubation in primary antibodies; EpCAM, Ki67, and GFP antibodies for 1 h at RT, and ESR1, PGR, and FOXA2 antibodies overnight at 4 °C. Tissues were washed thrice for 10 min each before incubation in appropriate species-specific Alexa Fluor-conjugated secondary antibodies (1:1000) for 40 min in the dark (Alexa Fluor 555, Cell Signaling Technology, Danvers, MA, cat # 4417; Alexa Fluor 568 Invitrogen, cat # A11036). After 2–10 min PBS washes, tissues were counterstained with DAPI (300 nM; BioLegend, San Diego, CA) and cover-slipped using fluoro-gel aqueous mounting media (Electron Microscopy Sciences, Hartfield, PA, cat # 17985–30). Omission of primary antibodies served as a negative control. Fluorescent imaging was performed using a Leica 5500 microscope.

### Blood serum collection and ELISAs

*Amhr2-Cre; Rosa-EYFP* and *Amhr2-Cre; Rosa26-tTA; H2B-GFP* females aged P21 were anesthetized prior to submandibular bleed. Blood was collected in 1.5-mL tubes, allowed to coagulate for 30 min to 1 h before centrifugation, 10,000 rpm at 4 °C for 10 min. Serum was removed, transferred into a clean Eppendorf tube, and stored at 20 °C until use. Mouse/rat estradiol and progesterone ELISA kits (Calibiotech, El Cajon, CA, cat #s ES180S-100 and PG362S, respectively) were used per manufacturer’s protocol to assess blood serum estradiol and progesterone concentrations and read using a Synergy HT multi-detection microplate reader (BioTek, Winooski, VT).

### Statistical analyses

All statistical analyses were performed using GraphPad Prism 9 software (San Diego, CA). One-way ANOVA with Tukey’s post-hoc test, Student’s *T*-test, and Pearson’s correlation coefficients were used where appropriate (indicated in figure legends) and significance was considered at *P* < 0.05.

## Results

### Mesenchymal-derived (MD) epithelial cells fluctuate across the estrous cycle in postpartum uteri

**Fig. 2:**
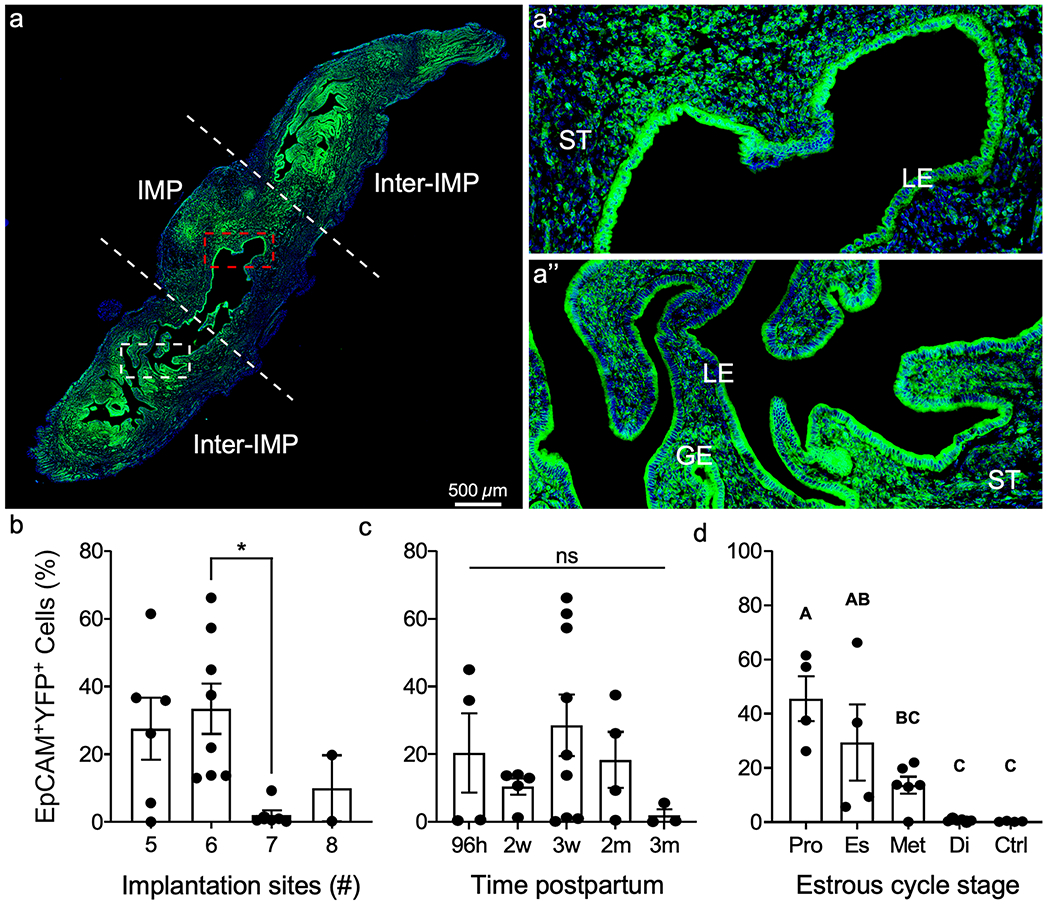
Mesenchymal-derived (MD) epithelial cells fluctuate across the estrous cycle in postpartum uteri. **a** Representative image of direct YFP fluorescence in a longitudinal section of a uterine horn following pregnancy and endometrial repair in *Amhr2-Cre; Rosa-EYFP* female mice. The implantation site (IMP) is demarcated by white dashed lines and is flanked by inter-implantation sites (Inter-IMP). **a’** Magnified image of red-boxed area in (**a**) showing YFP^+^ (MD) and YFP^−^ (non-MD) epithelial cells in the IMP site. **a”** Magnified image of white-boxed area in **a** showing YFP^+^ (MD) and YFP^−^ (non-MD) epithelial cells in the inter-IMP site. The percentages of EpCAM^+^YFP.^+^ (MD-epithelial cells) analyzed by flow cytometry were quantified and graphed by the number if implantation sites (**b**), time postpartum (**c**), and stage of the estrous cycle (**d**). Statistical analyses were performed by one-way ANOVA with significance at *P* < 0.05, indicated by * or different letters. ns, not significant; ST, stroma; LE, luminal epithelium; GE, glandular epithelium; EpCAM, epithelial cell adhesion molecule; YFP, yellow fluorescent protein; Pro, proestrus; Es, estrus; Met, metestrus; Di, diestrus; Ctrl, control (no cre)

Previous studies using lineage tracing mouse models to examine the fate of mesenchymal-derived (MD) cells identified MD-epithelial cells following postpartum and menses-like endometrial epithelial regeneration (Huang et al. 2012; Patterson et al. 2013). It was hypothesized that endometrial stromal-mesenchymal cells underwent MET as a mechanism to regenerate the epithelium following endometrial damage. MD-epithelial cells were present in the epithelium for at least 2 months postpartum (Huang et al. 2012), but additional evaluation of these cells was not conducted. Here, we sought to determine if the number of implantation sites, being the regions of greatest regeneration, was correlated with the number of MD-epithelial cells and if MD-epithelial cells increased over time postpartum. We used the Cre-lox system by crossing *Amhr2-Cre* mice with *Rosa26-Stop*^*fl/fl-EYFP*^ reporter mice to indelibly label uterine mesenchymal cells and any MD cells (Fig. 1a). As previously reported, the *Amhr2* promoter is only active in uterine mesenchymal cells but not in epithelial cells in embryonic, postnatal, and adult mice (Jamin et al. 2002; Arango et al. 2008; Huang et al. 2012; Patterson et al. 2013; Saatcioglu et al. 2019). Uteri were collected from *Amhr2-Cre; Rosa-EYFP* females at various time points postpartum after epithelial regeneration was completed, implantation sites were counted, and endometrial cells were analyzed by flow cytometry (gating strategy, SFig. 1a–a”) for EpCAM (epithelial cells) and EYFP (MD cells). The only significant difference between the number of implantation sites and the percentage of EpCAM^+^EYFP^+^ MD-epithelial cells was between six and seven implantation sites (Fig. 2b). Longitudinal sections of implantation sites and inter-implantation sites showed no qualitative differences in the presence of EYFP^+^ MD-epithelial cells (Fig. 2a–a”). There were no significant differences in the percentage of EpCAM^+^EYFP^+^ MD-epithelial cells present at 96 h, 2 weeks, 3 weeks, 2 months, and 3 months postpartum (Fig. 2c). The results showed extreme variability in the percentages of EpCAM^+^EYFP^+^ MD-epithelial cells postpartum. For example, with five implantation sites, the range in EpCAM^+^EYFP^+^ cells was 0.03 to 61.4%. Similar variability was also seen when analyzed by time postpartum. Subsequently, samples were categorized by the stage of the estrous cycle based on vaginal cytology. There was a significant decline in the percentage of EpCAM^+^EYFP^+^ cells from proestrus (47.35% ± 7.48%) to diestrus (0.81% ± 0.09%), with no difference between diestrus and control uteri (Fig. 2d). These data suggest that MD-epithelial cell populations fluctuate in response to hormonal changes across the estrous cycle irrespective of the number of sites of regeneration or time postpartum.

### MET occurs in the absence of endometrial damage and repair

**Fig. 3:**
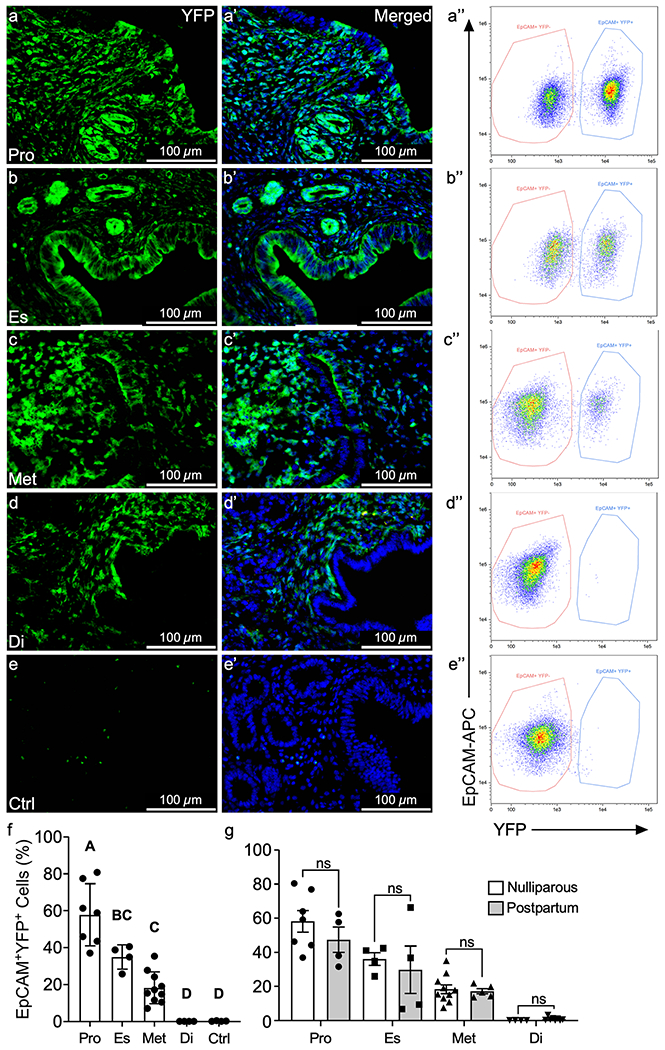
MET occurs in the absence of endometrial damage and repair. Representative images of direct YFP fluorescence in uterine cross sections from adult *Amhr2-Cre; Rosa-EYFP* female mice during proestrus (**a**, **a”**), estrus (**b**, **b”**), metestrus (**c**, **c”**), diestrus (**d**, **d”**), and control mice (no Cre) (**e**, **e”**). EpCAM^+^ YFP^−^ (non-MD) and EpCAM^+^YFP^+^(MD) epithelial cells were analyzed by flow cytometry in proestrus (**a”**), estrus (**b”**), metestrus (**c”**), diestrus (**d”**), and control (**e”**) with percentages of EpCAM^+^YFP^+^ cells graphed in (**f**). Percentages of EpCAM^+^YFP.^+^ MD-epithelial cells were compared by estrous cycle stage from virgin and postpartum mice (**g**). Statistical analyses were performed by one-way ANOVA (**f**) and *t*-test for each stage of the estrous cycle (**g**) with significance at *P* < 0.05 indicated by different letters. ns, not significant. EpCAM, epithelial cell adhesion molecule; Pro, proestrus; Es, estrus; Met, metestrus; Di, diestrus; Ctrl, control (no cre)

In previous studies, MD-epithelial cells were only observed after menses-like endometrial shedding or postpartum, but not in virgin mice (Huang et al. 2012). It was suggested that MET is reserved as a damage/repair mechanism of epithelial regeneration but is not required for minimal epithelial turnover during the estrous cycle. Although the endometrium is not shed and regenerated, as in the menstrual cycle, there is substantial growth and resorption that occurs across the short, 4–5 day estrous cycle in mice (Wood et al. 2007). Prior studies did not consider estrous cycle stage, and because of the significant differences in MD-epithelial cells seen across the cycle in postpartum uteri, we re-evaluated MET in virgin mice in each cycle stage. Similar to postpartum uteri, there was a high percentage of EpCAM^+^EYFP^+^ MD-epithelial cells (avg. 58%) identified in virgin uteri from *Amhr2-Cre; Rosa-EYFP* mice during proestrus that significantly declined to indistinguishable from control uteri in diestrus (Fig. 3f). Flow cytometry results were confirmed in uterine cross-sections, with direct visualization of YFP fluorescence (Fig. 3). When compared by cycle stage, EpCAM^+^EYFP^+^ MD-epithelial cells in postpartum and virgin uteri were statistically similar (Fig. 3g). These data suggest that MET can occur during the estrous cycle in virgin mice regardless of prior endometrial damage and repair (regeneration) events.

### MD-epithelial cells exhibit dynamic temporal expression patterns during postnatal epithelial remodeling

**Fig. 4:**
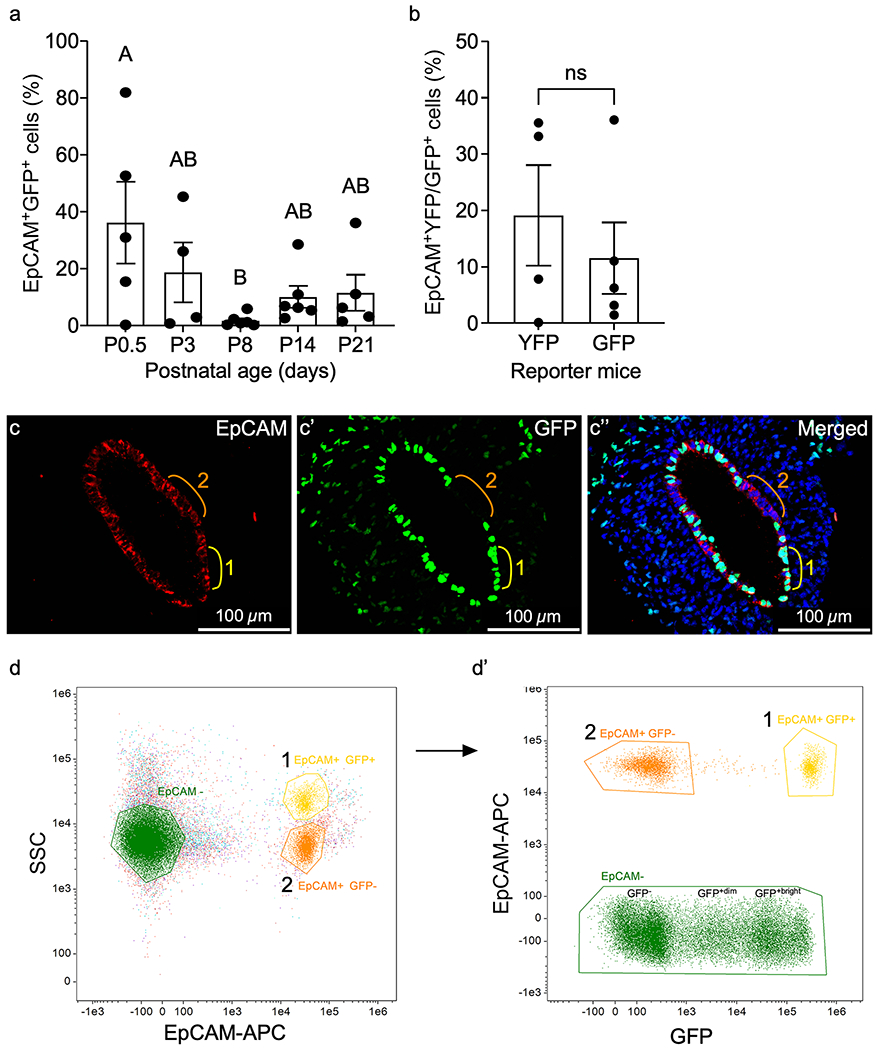
Mesenchymal-derived (MD) cells populate the epithelium during postnatal uterine maturation. (**a**) EpCAM^+^GFP^+^ (MD) epithelial cells from *Amhr2-Cre; Rosa26-tTA; H2B-GFP* mouse uteri at postnatal day (P) 0.5, P3, P8, P14, and P21 were analyzed by flow cytometry and graphed. (**b**) Graphical comparison of the percentages of endometrial EpCAM^+^YFP^+^ and EpCAM^+^GFP^+^ cells analyzed by flow cytometry from *Amhr2-Cre; Rosa-EYFP* and *Amhr2-Cre; Rosa26-tTA; H2B-GFP* female mice, respectively, at P21. (**c–c”**) Representative uterine cross section from an *Amhr2-Cre; Rosa26-tTA; H2B-GFP* mouse uterus at P0.5, corresponding to high % of EpCAM^+^GFP^+^ cells in (**a**), showing examples of EpCAM^+^GFP^−^ non-MD-epithelial cells (region 2, orange line), and EpCAM^+^GFP^+^ MD-epithelial cells (region 1, yellow line). Regions are represented in flow cytometry plots in (**d**, **d’**). (**d**) Representative flow cytometry plot from P0.5 mouse uterus of EpCAM^+^ epithelial cells that segregated into two populations (1 and 2) by side scatter (SSC). EpCAM-cells are also represented. (**d’**) EpCAM^+^ populations 1 and 2 from (**d**) were plotted by EpCAM and GFP expression. Population 1 with higher SSC in (**d**) was GFP^+^ in (**d’**) and population 2 with lower SSC in (**d**) was GFP^−^ in (**d’**). EpCAM^−^ cells from (**d**) are represented in (**d’**) and segregated into GFP^−^, GPF^+dim^, and GFP^+bright^. Statistical analyses were performed by one-way ANOVA (**a**) and *t*-test (**b**), with significance at *P* < 0.05 indicated by different letters. ns, not significant. EpCAM, epithelial cell adhesion molecule

Involvement of MD-epithelial cells in postnatal uterine maturation has not been previously explored. To investigate the temporal origin and possible contribution of MET to epithelial remodeling events postnatally, uteri were collected from *Amhr2-Cre; Rosa26-tTa; H2B-GFP* females (Fig. 1b) at postnatal days (P) 0.5, P3, P8, P14, and P21 to encompass epithelial expansion, adenogenesis, and differentiation. Flow cytometry data showed that EpCAM^+^GFP^+^ MD-epithelial cells arose rapidly after birth with high variability in samples ranging from 0.21 to 81.84% at P0.5 (and similar variability at P3), before declining significantly to P8, during initiation of adenogenesis (Fig. 4a). This variability may indicate dynamic turnover of the two epithelial cell populations leading up to adenogenesis, which is predominated by EpCAM^+^GFP^−^ non-MD-epithelial cells at P8 (Fig. 4a). Interestingly, EpCAM^+^GFP^+^ and EpCAM^+^GFP^−^ cells (visualized in Fig. 4c–c”, regions 1 and 2, respectively) segregated into two distinct populations by side scatter at P0.5 (Fig. 4d, d’, populations 1 and 2, respectively) and P3 (SFig. 2a, a’), indicating differences not only by GFP expression but also by cellular complexity between the two epithelial populations. The difference in cellular complexity was not present in later postnatal ages or in adult mice. It is also noted that two distinct EpCAM^−^GFP^+^ mesenchymal cell populations were revealed based on GFP intensity at P0.5 (Fig. 4d’) and P3 (SFig. 2a, a’). This may be indicative of different mesenchymal subpopulations as reported by Kirkwood et al. (2021). At P14, during the shift from adenogenesis to glandular morphogenesis (Vue et al. 2018; Vue and Behringer 2020), the percentage of EpCAM^+^GFP^+^ cells increased slightly, although not significantly, and was maintained through P21 (Fig. 4a). Some variability was noted at P21, with results from *Amhr2-Cre; Rosa-EYFP* females corroborating the same findings from *Amhr2-Cre; Rosa26-tTa; H2B-GFP* females at P21 (Fig. 4b). Depending on the strain (ours being on a mixed background), female mice reach puberty between 28 and 40 days of age as defined by a completed estrous cycle with ovulation (Bertolin and Murphy 2014; Pangas and Rajkovic 2014). However, low levels of ovarian production of estradiol (E_2_) are observed as early as P7 and then substantially increase after P20, and progesterone (P_4_) is detected around P10 and steadily increases to about P30 (Bell 2018) all prior to puberty. Because E_2_ and P_4_ are present prior to puberty, we sought to determine if variations in MD-epithelial cells at P21 correlated with hormones like in the adult. We assayed serum E_2_ and P_4_ concentrations at P21 and show no significant correlation with the percentage of MD-epithelial cells in *Amhr2-Cre; Rosa-EYFP* or *Amhr2-Cre; Rosa26-tTa; H2B-GFP* females (Tables 1 and 2, respectively). Together, these data suggest that epithelial cells originate from the mesenchyme very early after birth, presumably through MET, and are quickly replaced by non-MD-epithelial cells during adenogenesis. However, they return during gland morphogenesis and differentiation at P14 and are maintained, likely independently of ovarian E_2_ and P_4_ at P21.

### MD-epithelial cells are unique but have general characteristics of functional epithelial cells

**Fig. 5:**
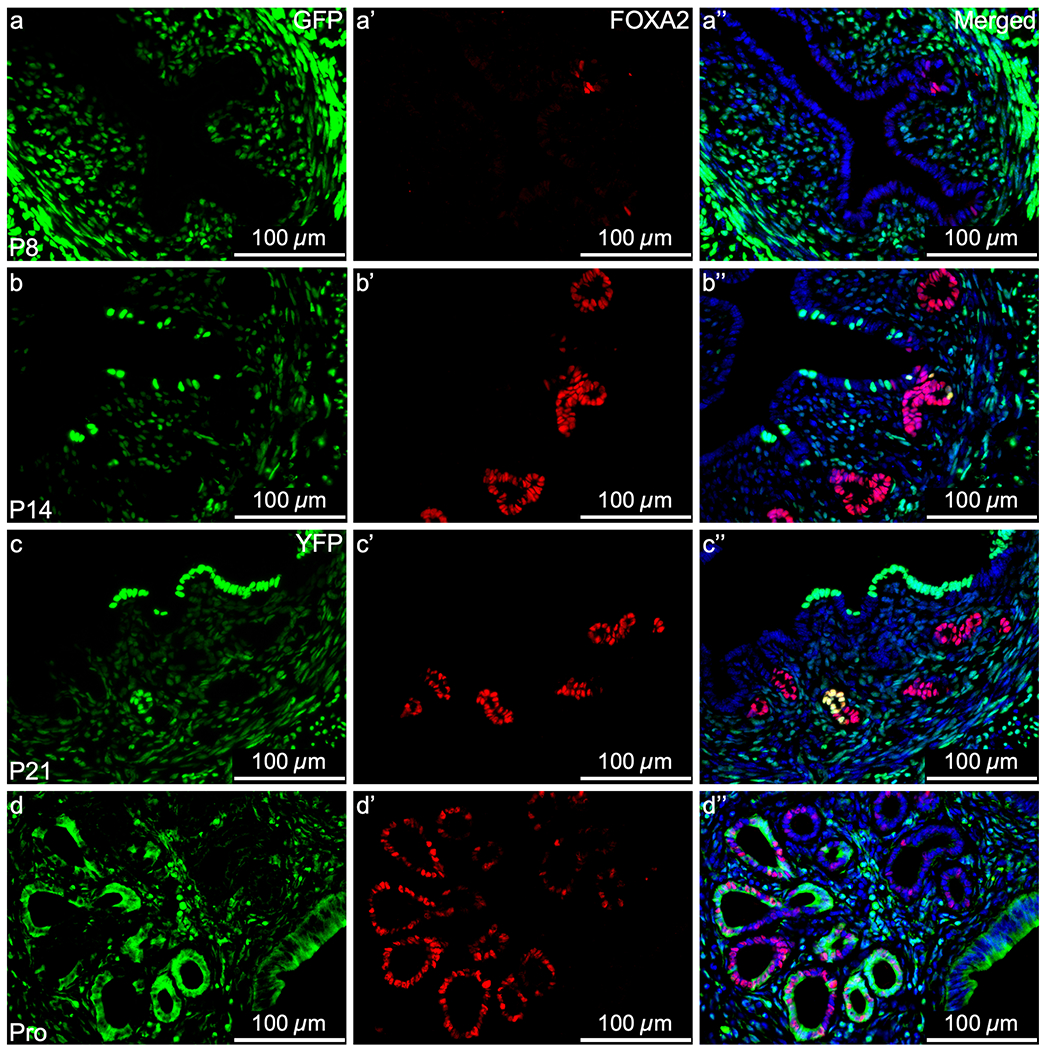
Mesenchymal-derived (MD) glandular epithelial cells express FOXA2. Representative images of uterine cross sections from *Amhr2-Cre; Rosa26-tTA; H2B-GFP* mice at postnatal day (P) 8 (**a–a”**), P14 (**b–b”**), P21 (**c–c”**), and from *Amhr2-Cre; Rosa-EYFP* mice in proestrus (Pro) (**d–d”**). (**a**, **b**, **c**, **d**) direct GFP/YFP expression in mesenchymal cells an MD-epithelial cells. (**a’**, **b’**, **c’**, **d’**) FOXA2 expression (red) by immunofluorescence, restricted to the glandular epithelium. (**a”**, **b”**, **c”**, **d”**) Merged images of the first two panels with nuclear DAPI staining (blue). FOXA2, Forkhead Box A2

We have shown that in addition to endometrial regeneration, MD-epithelial cells contribute to epithelial remodeling postnatally and in virgin mice. Due to the high turnover of MD-epithelial cells or replacement by non-MD-epithelial cells during postnatal uterine maturation and the estrous cycle, we investigated their epithelial characteristics to determine if they have the potential to function as *bone fide* endometrial epithelial cells. MD-epithelial cells expressed the epithelial marker, EpCAM, at all postnatal and adult time points (SFig. 1c–f, respectively). Importantly, EpCAM was specific to LE and GE, indicating mesenchymal cells were not inadvertently analyzed by flow cytometry. FOXA2, a marker of GE, was first observed at P8 (adenogenesis initiation), at which time point few cells showed nuclear expression (Fig. 5a–a”). Because very few GFP^+^ MD-epithelial cells were found at this time, subsequently, no co-expression of GFP and FOXA2 was observed in the GE. From P14 through adulthood, FOXA2 was expressed in the GE by both GFP^+^ MD-epithelial cells and GFP^−^ non-MD-epithelial cells similarly (Fig. 5b–d”).

**Fig. 6:**
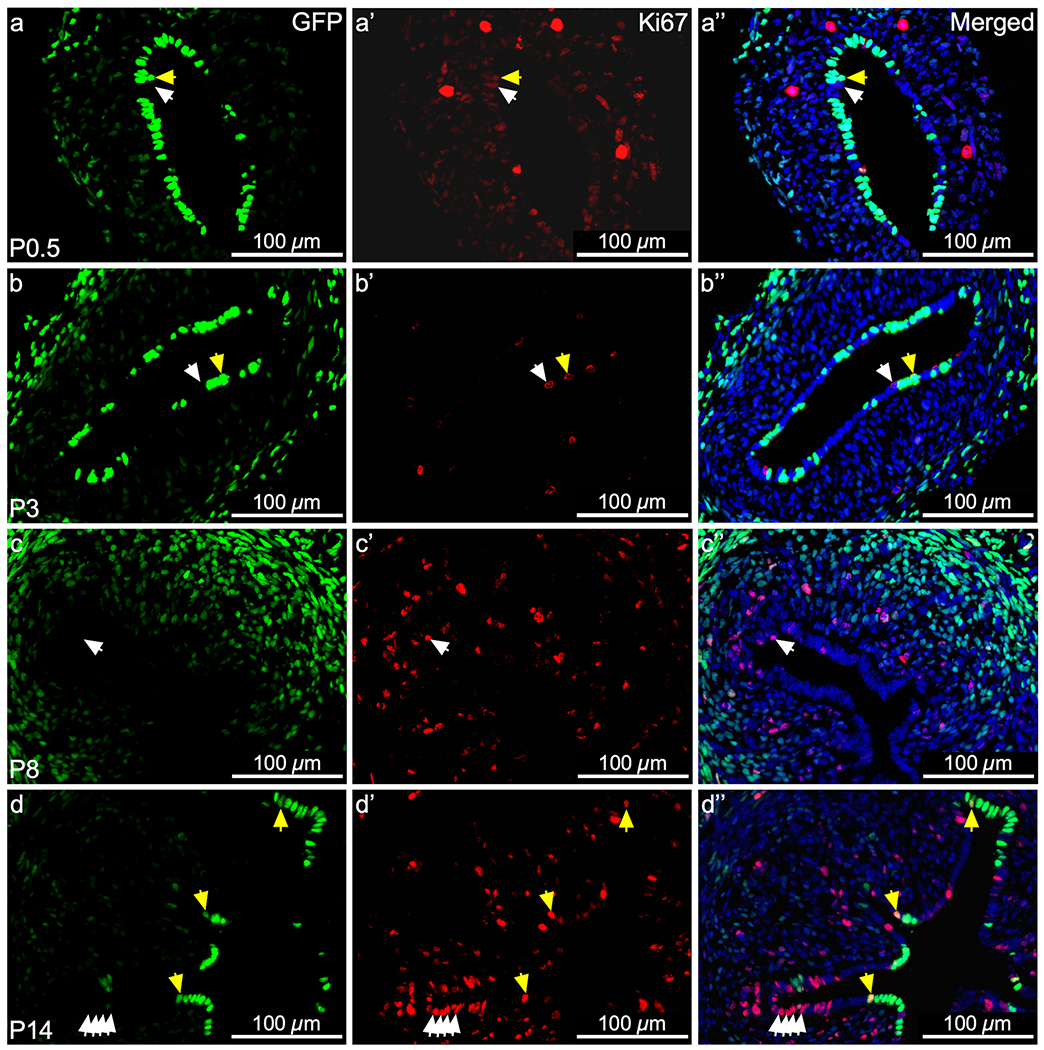
Mesenchymal-derived (MD) epithelial cells differentially contribute to epithelial remodeling by proliferation during postnatal uterine maturation. Representative images of uterine cross sections from *Amhr2-Cre; Rosa26-tTA; H2B-GFP* mice at postnatal day (P) 0.5 (**a–a”**), P3 (**b–b”**), P8 (**c–c”**), and P14 (**d–d”**). (**a**, **b**, **c**, **d**) Direct GFP expression in mesenchymal cells and MD-epithelial cells. (**a’**, **b’**, **c’**, **d’**) Ki67 expression (red) by immunofluorescence, indicating cells that proliferated. (**a”**, **b”**, **c”**, **d”)** Merged images of the first two panels with nuclear DAPI staining (blue). White arrow heads indicate GFP^−^ (non-MD) epithelial cells that expressed Ki67. Yellow arrow heads indicate GFP.^+^ (MD) epithelial cells that expressed Ki67

During postnatal maturation, initially, there was high proliferation at P0.5 with comparable expression of Ki67 in GFP^+^ MD-epithelial cells and GFP^−^ non-MD-epithelial cells in the undifferentiated LE (Fig. 6a–a”). At P3, there was an overall decline in expression of Ki67. Both GFP^+^ and GFP^−^ LE cells expressed Ki67 but it was slightly more prevalent in the GFP^−^ cells (Fig. 6b–b”). At P8 during adenogenesis initiation, there was minimal Ki67 expression in the epithelium which was largely devoid of GFP^+^ MD-epithelial cells (both GE and LE) (Fig. 6c–c”). By P14, LE proliferation was abundant and GFP^+^ MD-epithelial cells were again present, but the GE showed very few GFP^+^ cells and little proliferation. Expression patterns of Ki67 were distinct between GFP^+^ and GFP^−^ LE cells at this time. There were clusters of several GFP^−^ non-MD-epithelial cells that expressed Ki67, whereas typically, a single GFP^+^ MD-epithelial cell expressing Ki67 was followed by several more GFP^+^ cells that were negative for Ki67 (Fig. 6d–d”). At P21, there was little-to-no proliferation throughout the uterus (data not shown). Together, these results suggest that a divergence occurs between GFP^+^ and GFP^−^ epithelial cells around P3, such that GFP^+^ MD-epithelial cells are mostly absent at P8, return by P14, and there is preferential proliferation in GFP^−^ cells at P14, particularly in the LE.

**Fig. 7:**
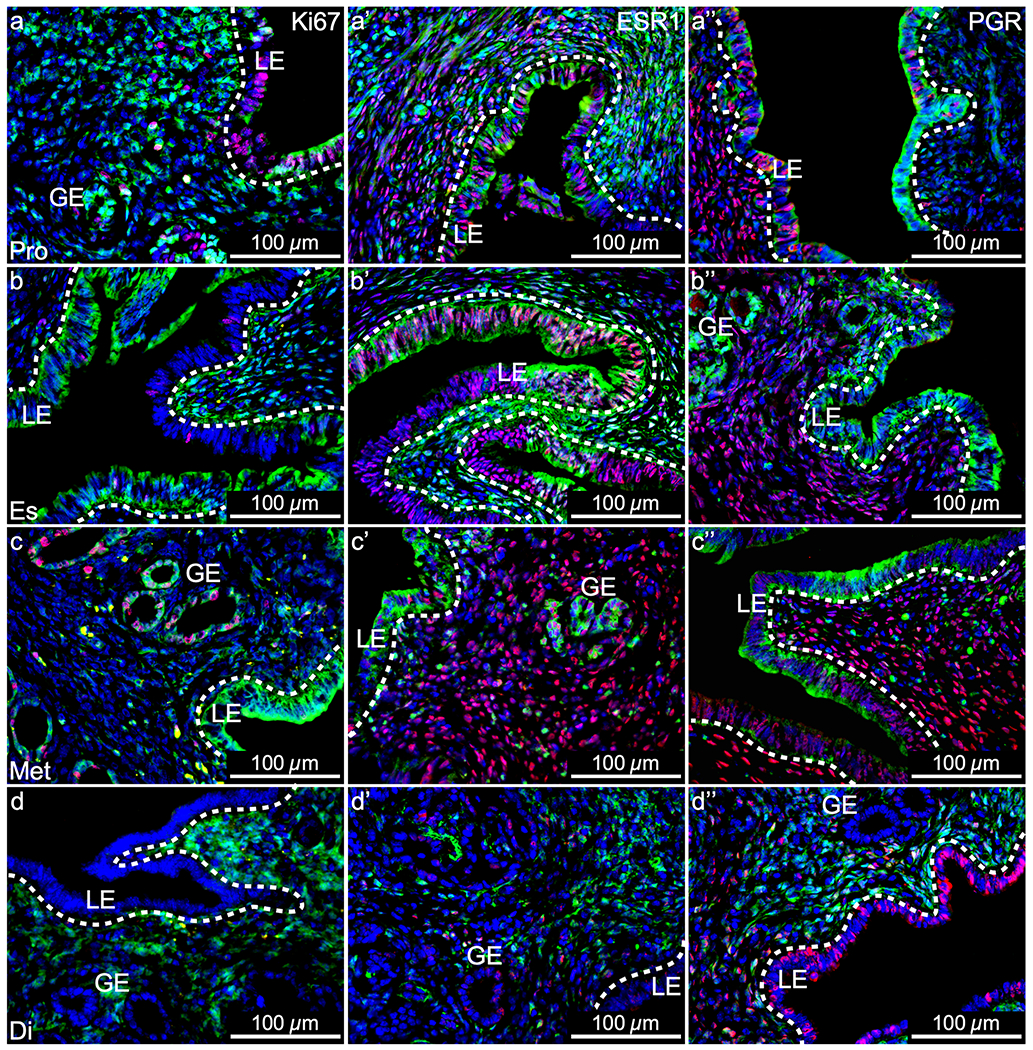
Mesenchymal-derived (MD) epithelial cells in adult uteri show characteristics of endometrial epithelial cells but are unique. Representative images of uterine cross sections from adult *Amhr2-Cre; Rosa26-EYFP* mice in proestrus (Pro) (**a–a”**), estrus (Es) (**b–b”**), metestrus (Met) (**c–c”**), and diestrus (Di) (**d–d”**). (**a**, **b**, **c**, **d**) Ki67 expression by immunofluorescence (IF, red), direct GFP expression in mesenchymal cells an MD-epithelial cells (green), and nuclear DAPI stain (blue). (**a’**, **b’**, **c’**, **d’**) ESR1 expression by IF (red), direct GFP expression (green), and nuclear DAPI stain (blue). (**a”**, **b”**, **c”**, **d”**) PGR expression by IF (red), direct GFP expression (green), and nuclear DAPI stain (blue). ESR1, estrogen receptor alpha; PGR, progesterone receptor; LE, luminal epithelium; GE, glandular epithelium; dotted lines demarcate LE from the underlying stroma

During the estrous cycle, Ki67, ESR1, and PGR expression varies dynamically from stage to stage (Wang et al. 2000; Mote et al. 2006). In proestrus, estrus, and metestrus, when YFP^+^ MD-epithelial cells were present, there appeared to be no qualitative differences in ESR1 and PGR expression compared to YFP^−^ non-MD-epithelial cells (Fig. 7, middle and right panels and SFigs. 4 and 5, respectively). Both YFP^−^ and YFP^+^ LE cells proliferated in response to rising estrogen in proestrus, indicated by Ki67 expression (Fig. 7a, SFig. 3a–a’). In estrus, there was an overall decrease in Ki67 expression in the LE and this appeared to be more evident in YFP^+^ compared to YFP^−^ cells (Fig. 7b, SFig. 3b–b’). By metestrus, proliferation was restricted to the GE and was similar in YFP^+^ and YFP^−^ cells (Fig. 7c, SFig. 3c–c’). In diestrus, there was a lack of epithelial proliferation and YFP^+^ MD-epithelial cells were absent (Fig. 7d, SFig. 3d–d’). These data support that MD-epithelial cells have endometrial epithelial characteristics. However, a distinction exists between MD-epithelial cells and non-MD-epithelial such that the former is replaced by the latter as the cycle progresses from proestrus to diestrus.

## Discussion

Prior research using *Amhr2-Cre* lineage tracing models showed that MD-cells populated the epithelium (GE and LE) following postpartum endometrial repair. These cells were not observed in pre/peri-pubertal (Patterson et al. 2013) or virgin (Huang et al. 2012) mice. Also, using a menses-like mouse model, putative mesenchymal-epithelial transitional cells that co-expressed vimentin and cytokeratin were identified in the regenerating endometrial stroma (Patterson et al. 2013; Cousins et al. 2014). These cells increased in number and appeared to migrate from the stromal-myometrial border to the repairing luminal epithelium as time progressed (Patterson et al. 2013). It was thus hypothesized that endometrial stromal-mesenchymal cells underwent MET as a mechanism of epithelial regeneration, and that this mechanism was reserved for damage/repair processes that occurred postpartum or during menstruation. However, in the current study, MD-epithelial cells were identified during postnatal uterine maturation and throughout the estrous cycle in virgin mice, both physiological processes that are not associated with damage and repair. We propose two explanations for the discrepancy in these reports regarding the occurrence of MET outside of damage/repair events in the endometrium. First, the current study used image-based flow cytometry which is a more sensitive technique for identifying MD-epithelial cells (EpCAM^+^YFP/GFP^+^). We show that except for two mice, the average percentage of MD-epithelial cells at P14 (*n* = 5) and P21 (*n* = 4) was around 6% which is difficult to observe in tissue sections. This could explain why Patterson et al. (2013) did not identify MD-epithelial cells at P14 and P25. Huang et al. (2012) evaluated virgin mice and did not observe MD-epithelial cells. However, they did not evaluate mice according to estrous cycle stage. We show that MD-epithelial cells are not detectable in mice in diestrus. Female mice are known to enter anestrous or prolonged diestrus when group-housed in the absence of a male (or their pheromones) (Whitten 1956, 1958; Lamond 1959). We speculate that the virgin mice (*n* = 5) assessed by Huang et al. (2012) were in diestrus or perhaps acyclic. Therefore, the current study does not negate the findings in previous reports that MET is a mechanism of epithelial repair but that it also occurs during other important epithelial remodeling events not associated with damage and repair. However, because of the rapid turnover of MD-epithelial cells during the estrous cycle regardless of prior damage/repair events, further analyses are needed during the initial hours of endometrial repair (postpartum/menstruation) to determine the direct contribution of these cells to epithelial regeneration.

MET as a mechanism of endometrial epithelial regeneration was recently challenged. In a study using the same Cre model with *LacZ* report (*Amhr2-Cre;Rosa26-LacZ*), *LacZ*^+^ cells were identified in the epithelium of adult virgin mice (Ghosh et al. 2020) consistent with what we show here. However, the authors concluded that the *LacZ*^+^ epithelial cells were not stromal-derived by MET, but rather originated embryonically from *Amhr2*-expressing coelomic epithelial (CE) cells (Ghosh et al. 2020). During embryonic development, a few CE cells are specified to become Müllerian duct epithelial (MDE) cells, which then invaginate into the mesenchyme and proliferate forming the epithelial tube of the Müllerian duct. The MDE in turn becomes the uterine epithelium after birth and in the adult (Klattig and Rnglert 2007). Ghosh et al. (2020) showed, similar to others (Arango et al. 2008), that the CE expresses *Amhr2* (indicating promoter activity) and thus concluded that *Amhr2*-expressing CE cells were the source of *LacZ*^+^ cells in the adult epithelium (Ghosh et al. 2020). However, this interpretation is incorrect because the CE does not express *Amhr2* until after invagination of the MDE, and the CE does not further contribute to the growing MDE after this stage (Guioli et al. 2007; Orvis and Behringer 2007; Arango et al. 2008). Of note, the MDE was negative for *Amhr2* expression/promoter activity in that study (Ghosh et al. 2020) as well as others (Jamin et al. 2002; Arango et al. 2008; Saatcioglu et al. 2019). Therefore, embryonic CE or MDE cells are not the source of *Amhr2*-lineage traced cells in postnatal and adult uterine epithelium. Furthermore, *Amhr2* promotor activity has been investigated postnatally and in adults. At no point during postnatal uterine maturation, in virgin adults, or during endometrial regeneration (postpartum and menses-like model) have uterine epithelia demonstrated *Amhr2* promotor activity (Arango et al. 2008; Huang et al. 2012; Patterson et al. 2013; Saatcioglu et al. 2019), supporting that reporter-labeled epithelial cells in *Amhr2-Cre* lineage tracing models are mesenchyme-derived.

The regulation of MET in endometrial remodeling is still under investigation; however, our current data provide insight into possible hormonal regulation in the adult. We show that MD-epithelial cells may arise by MET due to increasing E_2_, as the highest percentages were observed in proestrus and estrus. This is somewhat substantiated by a report of MET in vitro. Cultured mouse endometrial stromal cells were shown to transition to epithelial cells after 10 days of E_2_ treatment (Yin et al. 2019). Following a decline in E_2_ after ovulation, the MD-epithelial population in diestrus was indistinguishable from control (no Cre) uteri. Interestingly, the percentage of MD-epithelial cells increased from negligible in diestrus to comprising 30–80% of the epithelia in proestrus. Since the estrous cycle in mice is 4–5 days long, these data indicate that there is rapid and robust contribution from the stroma to epithelial expansion in no more than 1-day time. This may be due to a combination of rising E_2_ and a release from P_4_ inhibition. The MD-epithelial cells were then cleared or replaced in an exceptionally short amount of time, as well, as the cycle progressed back to diestrus. How MD-epithelial cells are “cleared” so quickly is under investigation. Apoptosis occurs during the cycle in the LE and GE. However, at the highest rates of apoptosis during metestrus, only ~ 2.5% of GE and ~ 27% of LE cells were apoptotic (Wood et al. 2007). This suggests there may be other mechanisms involved in replacement of the MD-epithelial cells. It is also unclear, at this time, how MD and non-MD epithelial cells are differentially regulated by E_2_ and P_4_ as they express ESR1 and PGR similarly. The uterus is proposed to contain epithelial stem/progenitor cells (Spooner et al. 2021). Results from the current study would suggest that both epithelial progenitors and stromal cells contribute to epithelial remodeling in the uterus because not all epithelial cells are mesenchymal-derived. Since MD-epithelial cells were cleared quickly across the estrous cycle, they may be replaced by epithelial progenitor cells.

We show that uterine MD-epithelial cells were present right after birth. Four out of five pups showed GFP^+^ uterine epithelial cells (range: 15–80%) at P0.5. Because these cells do not originate from the embryonic epithelium (discussed above), we speculate that they arise immediately after birth by MET. At birth, fetuses experience a sudden change in their hormonal exposure. The gestational environment is dominated by P_4_ produced by the mother, with P_4_ withdrawal occurring when the fetuses are removed from the uterine environment at birth. In humans, some newborn females experience uterine bleeding similar to that seen during menstruation in adults, and this has been suggested to be due to P_4_ withdrawal (Ober and Bernstein 1955; Puttemans et al. 2017). We speculate the MD-epithelial cells arise rapidly after birth influenced by P_4_ withdrawal. E_2_ is not required for postnatal epithelial remodeling (adenogenesis and differentiation), and uterine maturation remains ovarian steroid independent until approximately P25 (Ogasawara et al. 1983; Bigsby and Cunha 1985; Branham and Sheehan 1995). In line with this, at P21, our results showed no correlation between MD-epithelial populations and serum E_2_ or P_4_ concentrations. This contrasts with the adult where E_2_ and P_4_ regulate uterine function including epithelial remodeling. Because of this, MET in the postnatal uterus is likely mechanistically different from MET occurring in adult uteri. Further exploration during peripubertal time points is necessary to determine the temporal transition in MET regulation in the uterus. Insight might also be gleaned from information on mammary gland and kidney. Although MET specifically has not been demonstrated in the mammary gland, inhibition of EMT by the transcription factor Ovo-like 2 (OVOL2) is required for gland morphogenesis (development) and regeneration (Watanabe et al. 2014). In the developing kidney, cells of the metanephric mesenchyme undergo MET to form tubular epithelium (Horster et al. 1999). Bone morphogenetic protein 7 (BMP7) expression correlates with formation of the tubular epithelium from the metanephric mesenchyme (Luo et al. 1995; Vukicevic et al. 1996) and in the adult, exogenous administration of BMP7 induces MET in fibroblasts to facilitate repair of fibrotic kidney (Zeisberg et al. 2003a, b, 2005). Investigation into factors such as OVOL2 and BMP7 will be important for determining the regulation of MET in postnatal, as well as adult, epithelial remodeling.

Lastly, it should be noted that there is high variability in some of the data presented, particularly the postnatal results. We can speculate that the variability is due to the timing of tissue collection and development/maturation being a continuum (Vue et al. 2018). For example, at P8 during adenogenesis, some mice had distinguishable glands and others only had invaginations but not distinct glands. So, there is variability in the developmental status of the tissue at individual time points which could explain the variability in the results. This is also likely in the adult as the estrous cycle is a continuum as well. Therefore, collection in estrus may be early, mid, or late in the stage resulting in variability in the data. Moving forward, endometrial organoids may be used to recapitulate the uterine environment in an in vitro model since epithelial-stromal interactions are crucial to respond correctly to hormones. This will aid in investigating the regulation of MET in pre/peri-pubertal vs adult uteri in a more controlled environment with reduced variability. The 3D culture system will also be important for understanding of the role of MET in women.

In conclusion, this study has demonstrated using lineage tracing models that MET occurs during non-damage/repair epithelial remodeling events, contrary to previous beliefs. MD-epithelial cells were found during E_2_-dominant stages of the estrous cycle, as well as during postnatal maturation, suggesting MET makes a greater contribution to epithelial remodeling than solely being a mechanism for endometrial repair. In both postnatal maturation and during the estrous cycle, MD-epithelial cells exhibited rapid turnover. Despite this, MD-epithelial cells expressed similar markers to non-MD-epithelial cells, supporting that although distinct, these cells have gained epithelial characteristics during MET. Based on our data, we hypothesize that E_2_ may have a role in MET in the adult, but P_4_ withdrawal may contribute during ovarian steroid independent stages: the mechanism inducing MET is still unknown and requires further study. Future research will continue to characterize MD-epithelial cells with lineage tracing models, organoids, and begin to address the mechanism behind MET. This study provides important insight into MET as a mechanism of normal physiological endometrial remodeling under non-damage/repair conditions. This is relevant for further understanding of how MET when mis-regulated may contribute to endometrial disease or dysfunction.

## Supplementary Material

SFig. 1

SFig. 2

SFig. 3

SFig. 4

SFig. 5

## Figures and Tables

**Table 1:** Correlation between the percentages of EpCAM^+^YFP^+^ endometrial cells from *Amhr2-Cre; Rosa-EYFP* female mice with serum estradiol (E2) and progesterone (P4) concentrations at P21

% EpCAM^+^YFP^+^ cells	E2 concentration	P4 concentration
0.09	4.524	4.667
7.77	2.258	4.610
33.1	4.276	5.077
35.5	2.937	4.460
Pearson correlation	−0.065	0.212
*P*-value	0.966	0.788

**Table 2:** Correlation between the percentage of EpCAM^+^GFP^+^ endometrial cells with serum estradiol (E2) and progesterone (P4) concentrations at P21 from *Amhr2-Cre; Rosa26-tTA; H2B-GFP* female mice

% Epcam^+^GFP^+^ cells	E2 concentration	P4 concentration
1.43	3.728	1.806
3.13	7.368	3.615
6.22	3.362	2.978
10.99	2.346	2.700
36.01	2.486	4.010
Pearson correlation	−0.504	0.661
*P*-value	0.387	0.225
