# Supplementary figures and images for: A re-appraisal of mesenchymal-epithelial transition (MET) in endometrial epithelial remodeling
Source: Cell Tissue Res. Author manuscript; Available in PMC 2023 Feb 4. (PMC9889438; doi:10.1007/s00441-022-03711-z)

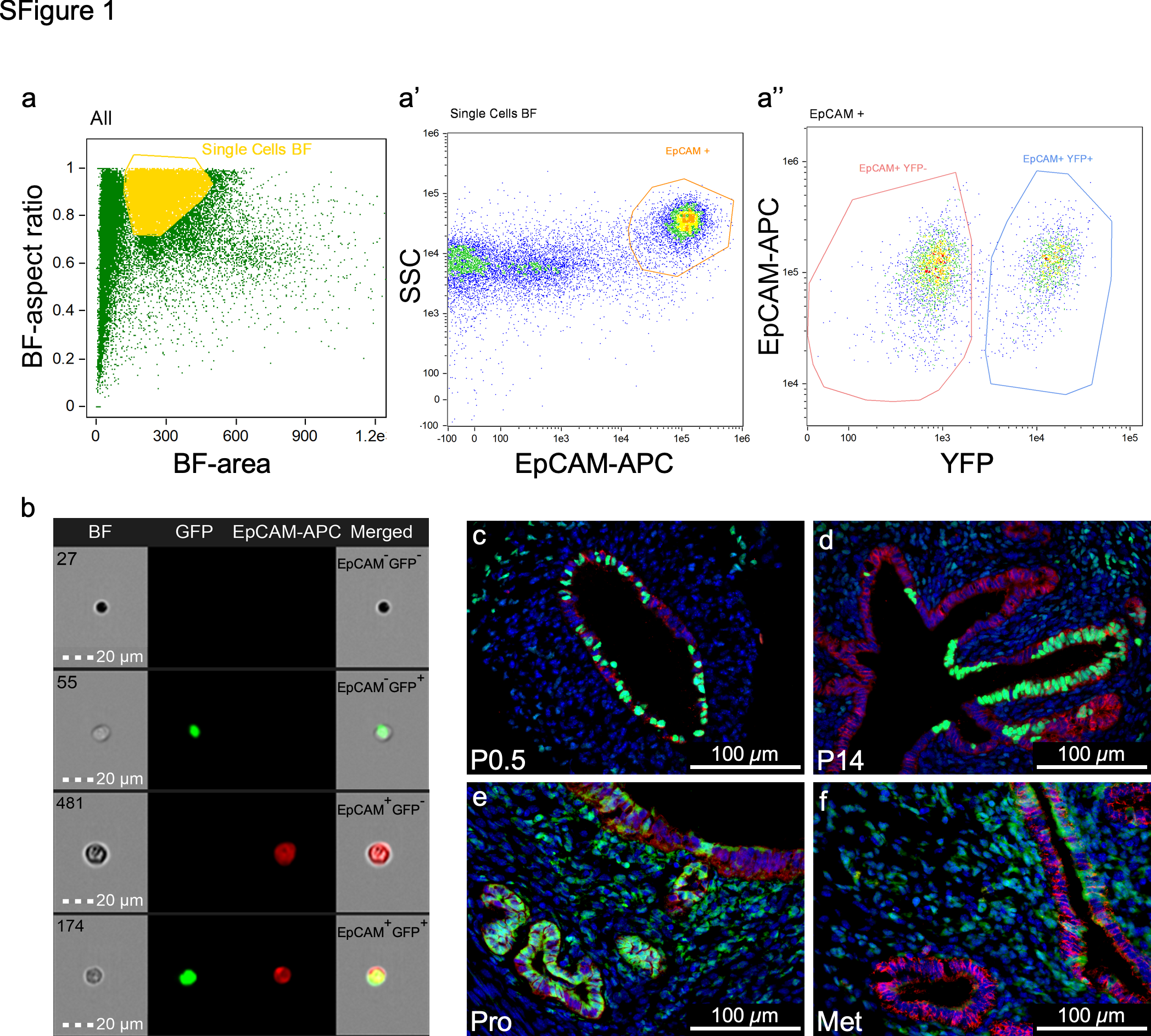

Supplement: SFig. 1 [file NIHMS1864147-supplement-SFig__1.tif]

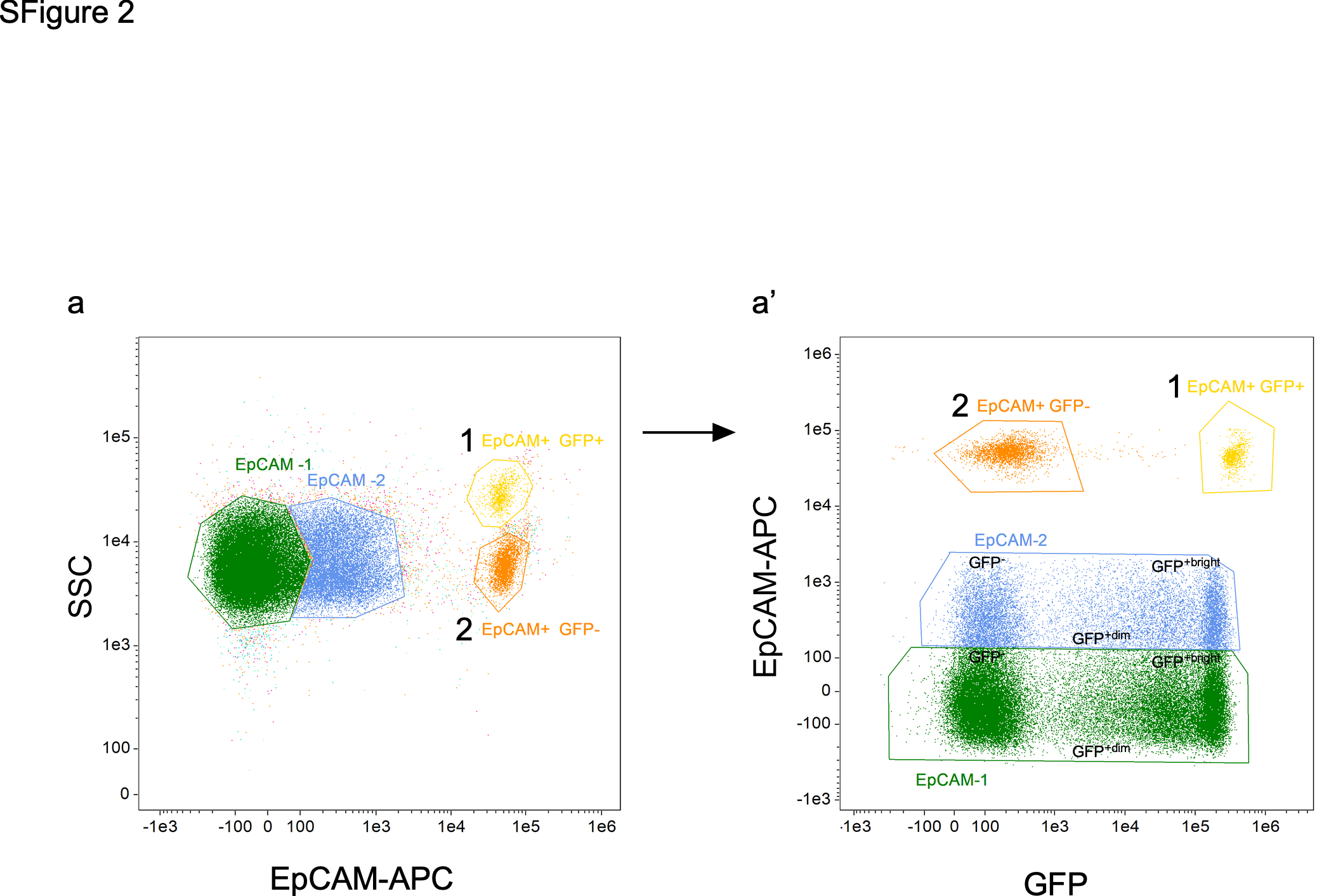

Supplement: SFig. 2 [file NIHMS1864147-supplement-SFig__2.tif]

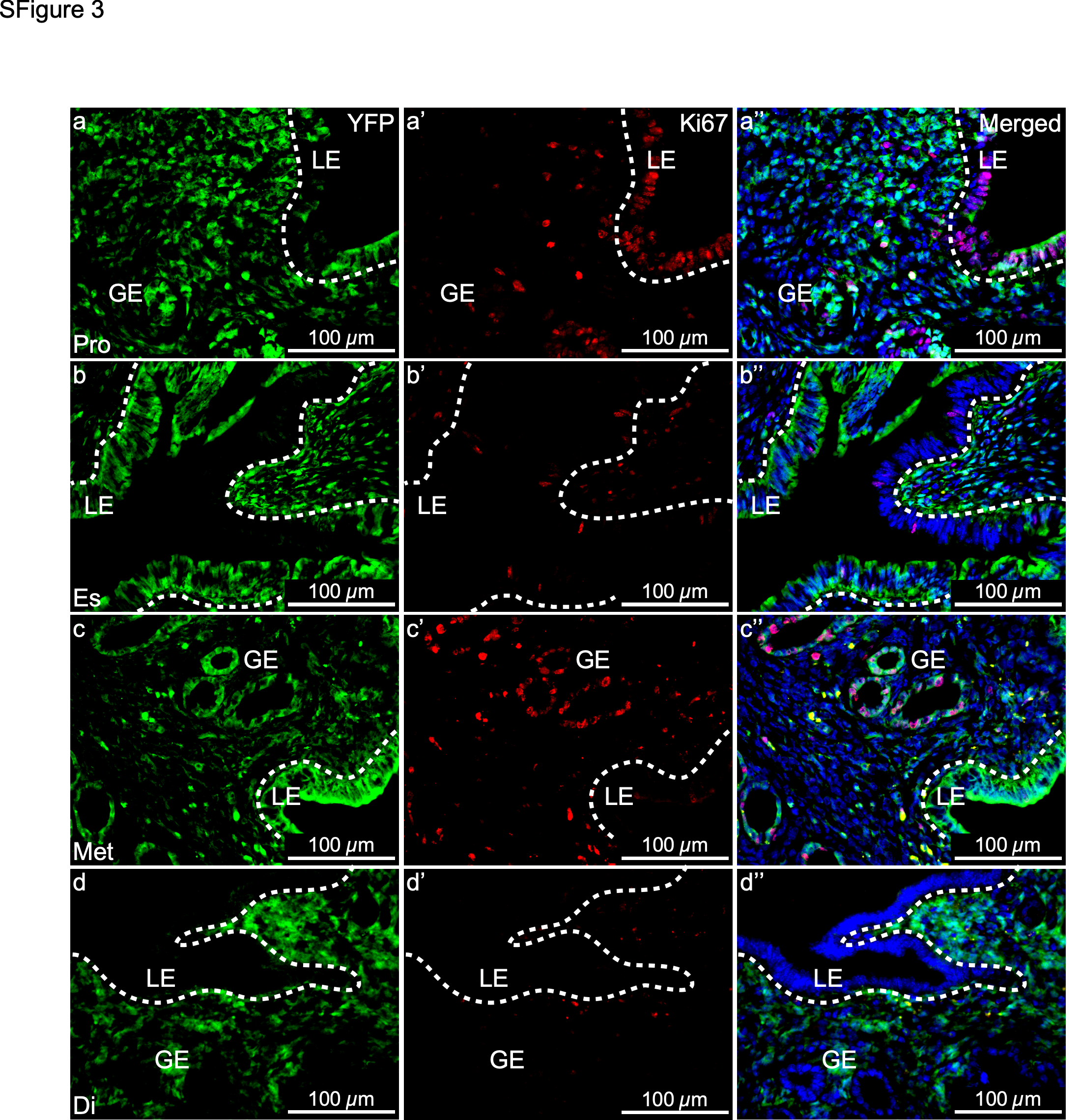

Supplement: SFig. 3 [file NIHMS1864147-supplement-SFig__3.tif]

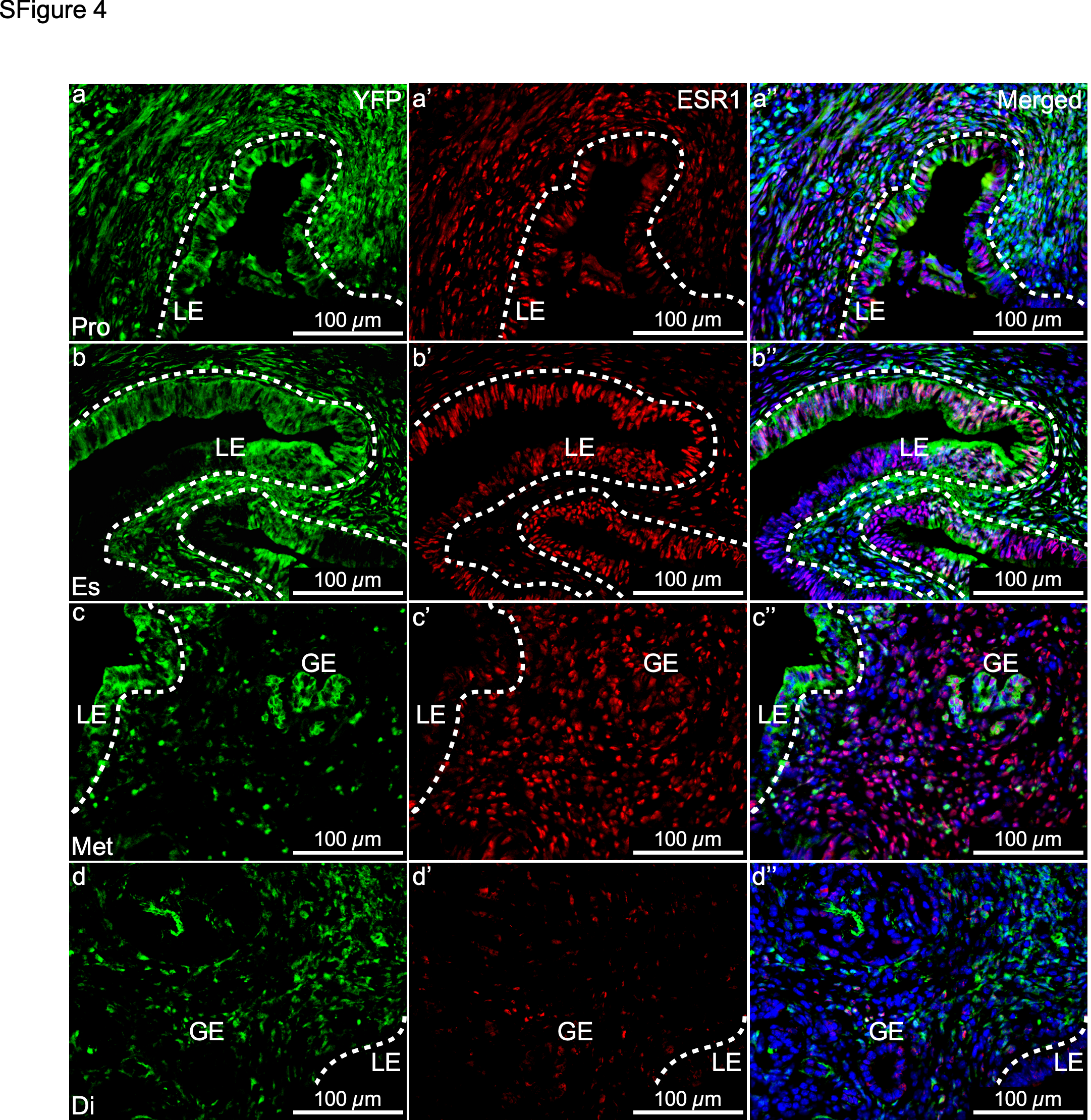

Supplement: SFig. 4 [file NIHMS1864147-supplement-SFig__4.tif]

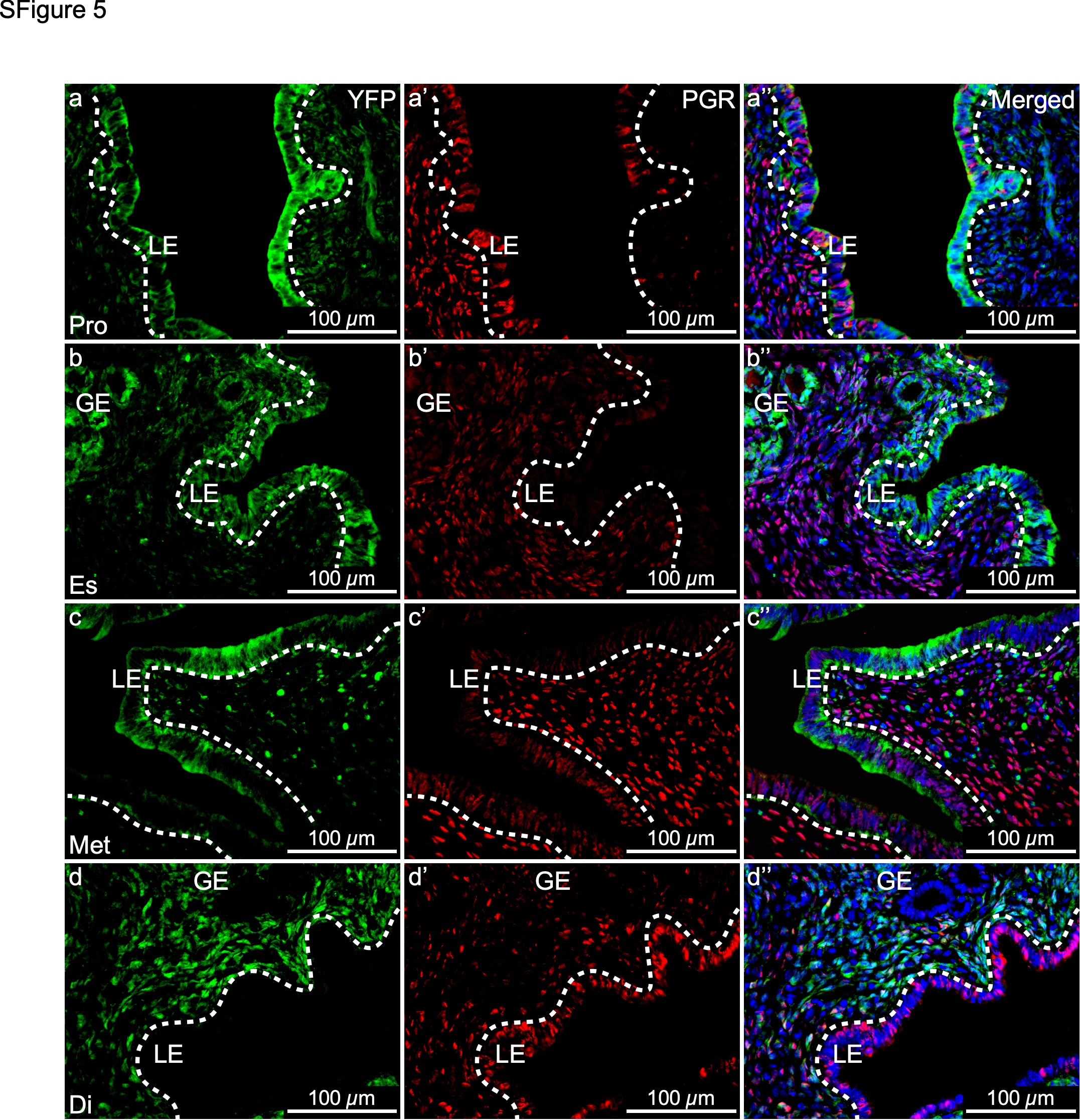

Supplement: SFig. 5 [file NIHMS1864147-supplement-SFig__5.tif]
